# Photovoltaic Performance of Pin Junction Nanocone Array Solar Cells with Enhanced Effective Optical Absorption

**DOI:** 10.1186/s11671-018-2727-7

**Published:** 2018-10-03

**Authors:** Jinnan Zhang, Lingmei Ai, Xin Yan, Yao Wu, Wei Wei, Mingqian Zhang, Xia Zhang

**Affiliations:** 1 State Key Laboratory of Information Photonics and Optical Communications, Beijing University of Posts and Telecommunications, Beijing, 100876 China; 2 School of Mechanical and Electric Engineering, Guangzhou University, Guangzhou, 510006 China; 3 Qian Xuesen Laboratory of Space Technology, China Academy of Space Technology, Beijing, 100094 China

**Keywords:** Nanocone array, Photovoltaic, Conversion efficiency, GaAs nanowire

## Abstract

The photovoltaic performance of axial and radial pin junction GaAs nanocone array solar cells is investigated. Compared with the cylinder nanowire arrays, the nanocone arrays not only improve the whole optical absorption but more importantly enhance the effective absorption (absorption in the depletion region). The enhanced effective absorption is attributed to the downward shift and extension of the absorption region induced by the shrinking top, which dramatically suppresses the absorption loss in the high-doped top region and enhances the absorption in the depletion region. The highest conversion efficiencies for axial and radial GaAs nanocone solar cells are 20.1% and 17.4%, obtained at a slope angle of 5° and 6°, respectively, both of which are much higher than their cylinder nanowire counterparts. The nanocone structures are promising candidates for high-efficiency solar cells.

## Background

Low-dimensional materials, including quantum dots (QDs), nanowires (NWs), and two-dimensional layered materials, are promising for photovoltaic applications due to their unique properties [1–5]. In comparison with their planar counterparts, III–V nanowire (NW) arrays have excellent optical properties such as anti-reflection and light trapping, showing great potential in high-performance solar cells [6–8]. Moreover, the ultrasmall footprint area of NWs sufficiently reduces the material consumption and increases the tolerance of lattice mismatch, enabling the realization of low-cost solar cells with less material and cheaper substrate [9–13]. The pin junction is the key part of a NW solar cell, which absorbs light and converts photons into electron-hole pairs. According to the pin junction geometry, NW array solar cells can be divided into axial and radial (or core-shell) pin solar cells, both of which have been widely investigated. However, up to date, the best efficiencies for axial and radial III–V NW array solar cells are 15.3% and 7.43%, respectively, still much lower than their planar counterparts [14, 15].

So far, many efforts have been made to improve the performance of NW array solar cells, mainly including the optimization of the diameter/period (*D*/*P*) ratio, diameter, and length, to obtain a better optical absorption of the whole NW arrays [16–20]. However, the absorption enhancement of the whole NW arrays does not necessarily lead to an increase of the ultimate conversion efficiency. As for practical NW pin arrays, the photocarriers generated in the p (or n) region quickly recombine due to lack of built-in electric field. Hence, to some extent, the absorption in the depletion region, or the effective optical absorption, directly determines the ultimate efficiency. However, for typical cylinder NW arrays, most of the light is absorbed by the upper part of NWs [16], while the absorption of the depletion region, which usually locates at the middle, is not sufficient. Particularly, for axial pin NW arrays, the incident light should pass through the p(n) region before absorbed by the depletion region, leading to great loss of light.

One possible way to enhance the effective optical absorption of NW arrays is to modulate the geometry of NW. For example, axial pin-inclined NW array solar cell has been reported to enhance the absorption of the depletion region by reducing the absorption of the top p (or n) region [21]. However, in practice, the *D*/*P* ratio should be much lower than the vertical NW arrays to avoid crossover of adjacent NWs, which limits the conversion efficiency. Tapered NWs, or nanocones, are expected to enhance the effective optical absorption as the incident light can be directly absorbed by the depletion region without passing through the top region. Up to date, nanocones with different slope angles and aspect ratios have been fabricated by Au-catalyzed vapor-liquid-solid and self-assembled catalyst-free methods [22–25], and the optical absorption properties have also been simulated [26, 27]. In practical solar cells, the influence of doping on the transport and optical properties cannot be ignored, and the radiative, Auger and Shockley-Read-Hall (SRH) recombination also plays an important role in the photoelectric conversion. However, to our knowledge, the photovoltaic performance of nanocone p(i)n solar cells considering the abovementioned factors has not been studied in detail yet.

In this paper, a coupled three-dimensional (3D) optoelectronic simulation is presented to investigate the photovoltaic performance of axial and radial pin junction GaAs nanocone solar cells. The optical absorption properties were investigated by using the finite-difference time-domain (FDTD). The photogeneration profiles were then incorporated into the electrical simulations to perform the calculation of the current density versus voltage (J-V) characteristics using finite element method (FEM). The doping-dependent mobility, bandgap narrowing, and radiative, Auger and SRH recombination were all taken into consideration in the electrical simulations. The highest efficiencies for axial and radial pin junction nanocone solar cells are 20.1% and 17.4%, respectively, much higher than their cylinder NW counterparts. The mechanism of the efficiency enhancement is discussed.

## Methods

**Fig. 1:**
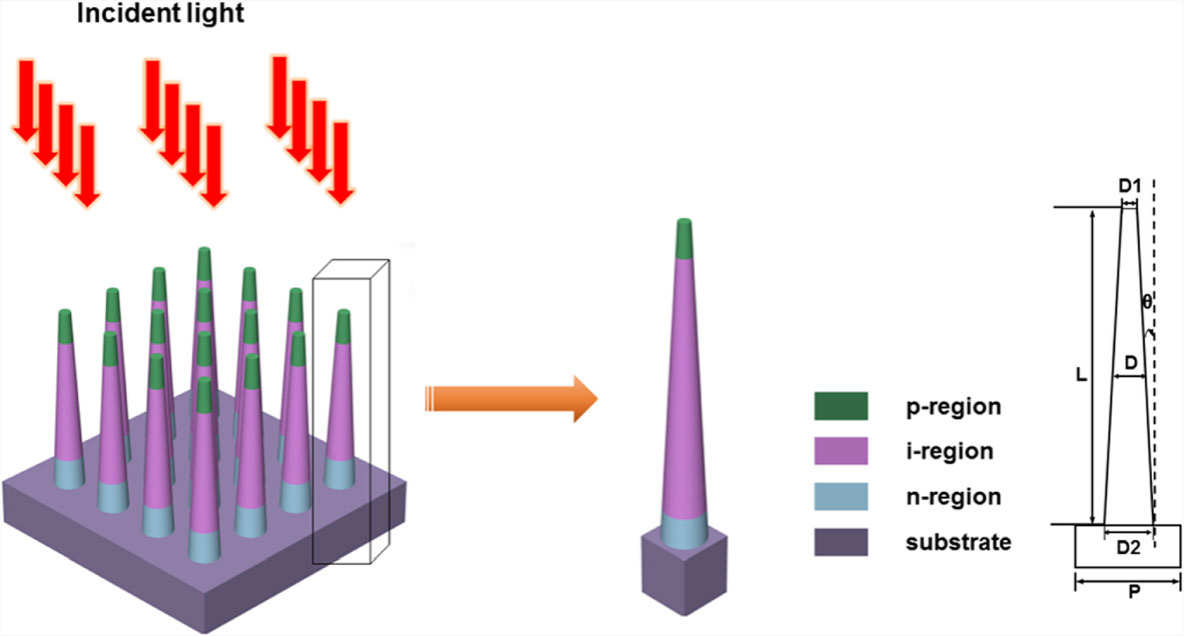
The schematic diagram of the axial pin junction GaAs nanocone arrays

The axial pin GaAs nanocone array model is shown in Fig. 1, which consists of periodic axial pin GaAs nanocones with diameter *D* = 180 nm, period *P* = 360 nm, and length *L* = 2 μm. Both the p- and n-regions have a length of 200 nm and are uniformly doped to 3 × 10^18^ cm^−3^ and 1 × 10^17^ cm^−3^, respectively. The GaAs substrate is n-doped with a carrier concentration of 1 × 10^17^ cm^−3^. The nanocone diameter is defined as the average of the top and bottom diameters. The slope angle (*θ*) is the angle between the sidewall and the normal direction of the bottom surface (substrate). In the simulation, the slope angle is changed from 0 to 5° by varying the bottom and top diameters while maintaining the average diameter constant.

Optical properties of the structure are investigated through Sentaurus Electromagnetic Wave (EMW) Solver module package [28–30]. The minimum cell size of the FDTD mesh is set to 5 nm, and the number of nodes per wavelength is 20 in all directions. By placing periodic boundary conditions, the simulations can be carried out in a single unit cell to model the periodic array structure. In order to save the resources and time required for the calculation, the thickness of the GaAs substrate is limited to 0.4 μm [21]. However, by using a perfect match layer (PML) adjacent to the GaAs substrate, the transmission light is totally absorbed, which enables us to model a semi-infinite GaAs substrate [31]. The wavelength-dependent complex refractive index used to describe the material dispersion properties of GaAs can be obtained from the study of Levinshtein et al. [32]. The incident light from the top is set in parallel to the NW axis as indicated in Fig. 1. We use a plane wave defined with power intensity and wavelength values from a discretized AM 1.5G solar spectrum with a wavelength ranging from 290 to 900 nm (typical absorption region of GaAs) to model the sunlight [33]. The total optical generation under AM 1.5G illumination can be modeled by superimposing the power-weighted single-wavelength optical generation rates [20]. The optical generation rate *G*_*ph*_ is obtained from the Poynting vector S:

1 $$ {G}_{ph}=\frac{\left|\overrightarrow{\nabla}\cdot \overrightarrow{S}\right|}{2\mathrm{\hslash}\omega }=\frac{\varepsilon^{{\prime\prime} }{\left|\overrightarrow{E}\right|}^2}{2\mathrm{\hslash}} $$

where *ħ* is the reduced Planck’s constant, *ω* is the angular frequency of the incident light, *E* is the electric field intensity at each grid point, and *ε*″ is the imaginary part of the permittivity. The reflection monitor is located above the top surface of the NWA, and the transmission monitor is located at the bottom surface of the substrate to calculate the light absorbed. The amount of power transmitted through the power monitors is normalized to the source power at each wavelength. The reflectance *R*(*λ*) and transmission *T*(*λ*) are calculated by the following equation:

2 $$ R\left(\lambda \right),T\left(\lambda \right)=0.5\int \mathrm{real}\left\{p{\left(\lambda \right)}_{\mathrm{monitor}}\right\} dS/{P}_{\mathrm{in}}\left(\lambda \right) $$

where *P*(*λ*) is the Poynting vector, *dS* is the surface normal, and *P*_in_(*λ*) is the incident source power at each wavelength. The absorption spectrum *A*(*λ*) of the GaAs NWAs is given by the following equation:

3 $$ A\left(\lambda \right)=1-R\left(\lambda \right)-T\left(\lambda \right) $$

For the electrical modeling, the 3D optical generation profiles are incorporated into the finite-element mesh of the NWs in the electrical tool, which solves the carrier continuity equations coupled with Poisson’s equation self-consistently in 3D. The doping-dependent mobility, bandgap narrowing, and radiative, Auger and SRH recombination are taken into consideration in the device electrical simulations. The critical material parameters for device simulations are mostly obtained from Levinshtein’s model [32], which is shown in Table 1.

**Table 1:** Key material parameters

Parameters	Electron (hole)
Minimum mobility	2.136 × 10^3^ (21.48) cm^2^/Vs
SRH lifetime	1 ns
Effective density of states	4.42 × 10^17^(8.47 × 10^17^)/cm^3^
Auger coefficient	1.9 × 10^−31^(1.2 × 10^−31^) (cm^6^/s)

## Results and Discussion

### Axial Pin Junction GaAs Nanocone Array Solar Cells

**Fig. 2:**
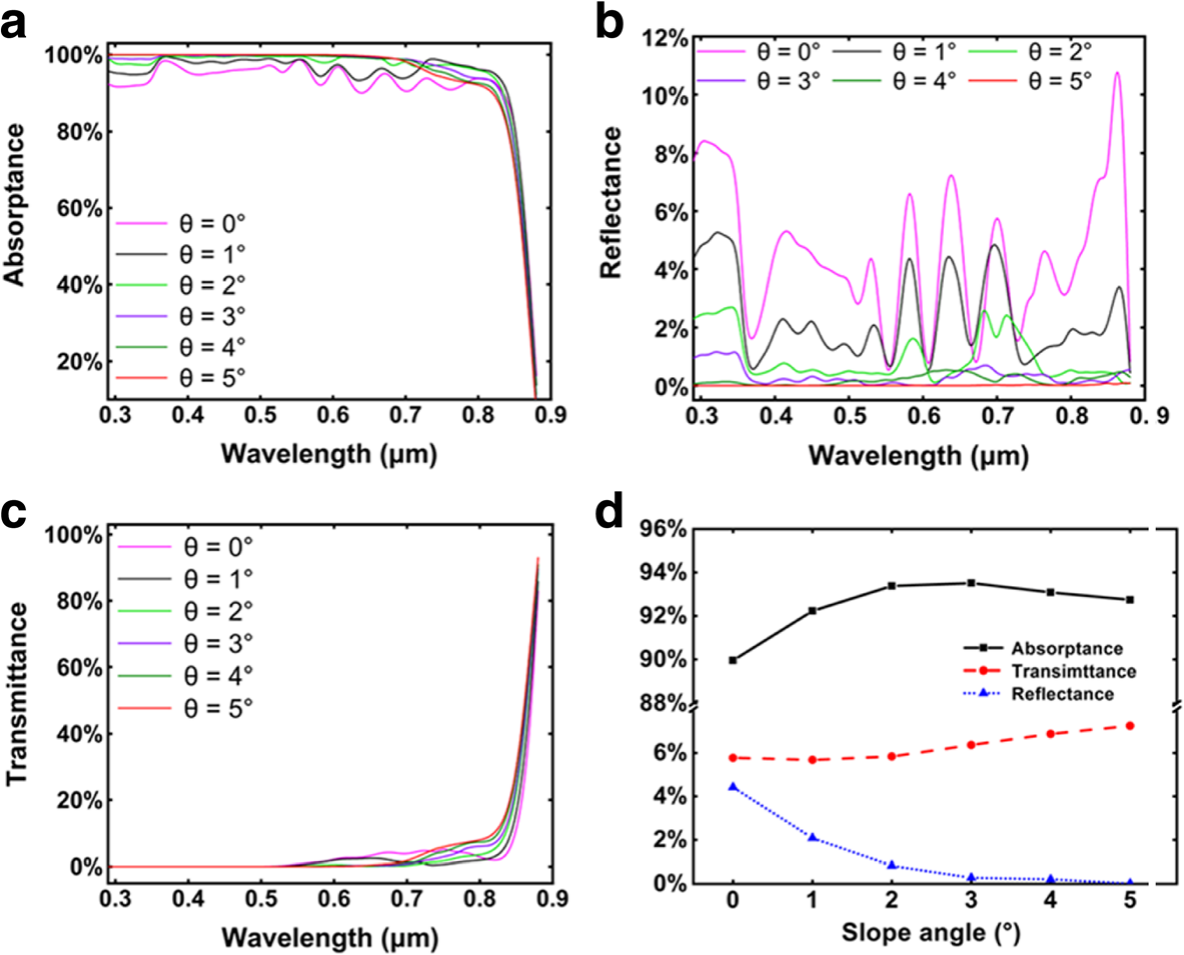
**a** Absorptance, **b** reflectance, and **c** transmittance of the axial pin junction GaAs nanocone arrays with *D*/*P* = 0.5 and *D* = 0.18 μm. **d** The AM1.5G-weighted integral of the absorptance, reflectance, and transmittance of the axial nanocone arrays with different slope angles

Figure 2a–c shows the wavelength-dependent absorptance, reflectance, and transmittance of the axial GaAs nanocone arrays with different slope angles. Compared with the cylinder NW arrays (*θ* = 0°), nanocone arrays show lower reflectance over the whole wavelength range, and the phenomenon becomes more obvious with the increase of slope angle. The anti-reflection ability of the NW arrays can be attributed to the low filling ratio, which reduces the effective refractive index and offers a good impedance match between GaAs and air [7]. For the nanocone arrays with a large slope angle, the filling ratio at the top of the arrays is extremely low, leading to nearly perfect impedance match with air and almost zero reflection. In the short wavelength range of 300–700 nm, the absorptance increases with the increase of slope angle due to the suppressed reflection. However, the absorptance of long wavelength light near GaAs bandgap decreases at large slope due to the very thin nanocone top which is not able to support optical modes. Figure 2d shows the AM 1.5G weighted integral of the absorptance, reflectance, and transmittance spectra for different slope angles. At small angles, the absorptance increases with increasing slope angle due to the decreased reflectance. When the slope angle exceeds 3°, the absorptance slightly decreases. This is probably attributed to the reduced absorption path as the very thin nanocone top is not able to support long wavelength modes. Nevertheless, the total absorption of nanocones at different slope angles (1~5°) has very little difference (in the range of 92~ 93.5%), suggesting that the slope angle has little influence on the total absorption of nanocones. Alternatively, the slope angle is believed to have a strong impact on the absorption in the intrinsic region, which dominates the photoelectric conversion efficiency. This will be discussed in detail in the following part.

**Fig. 3:**
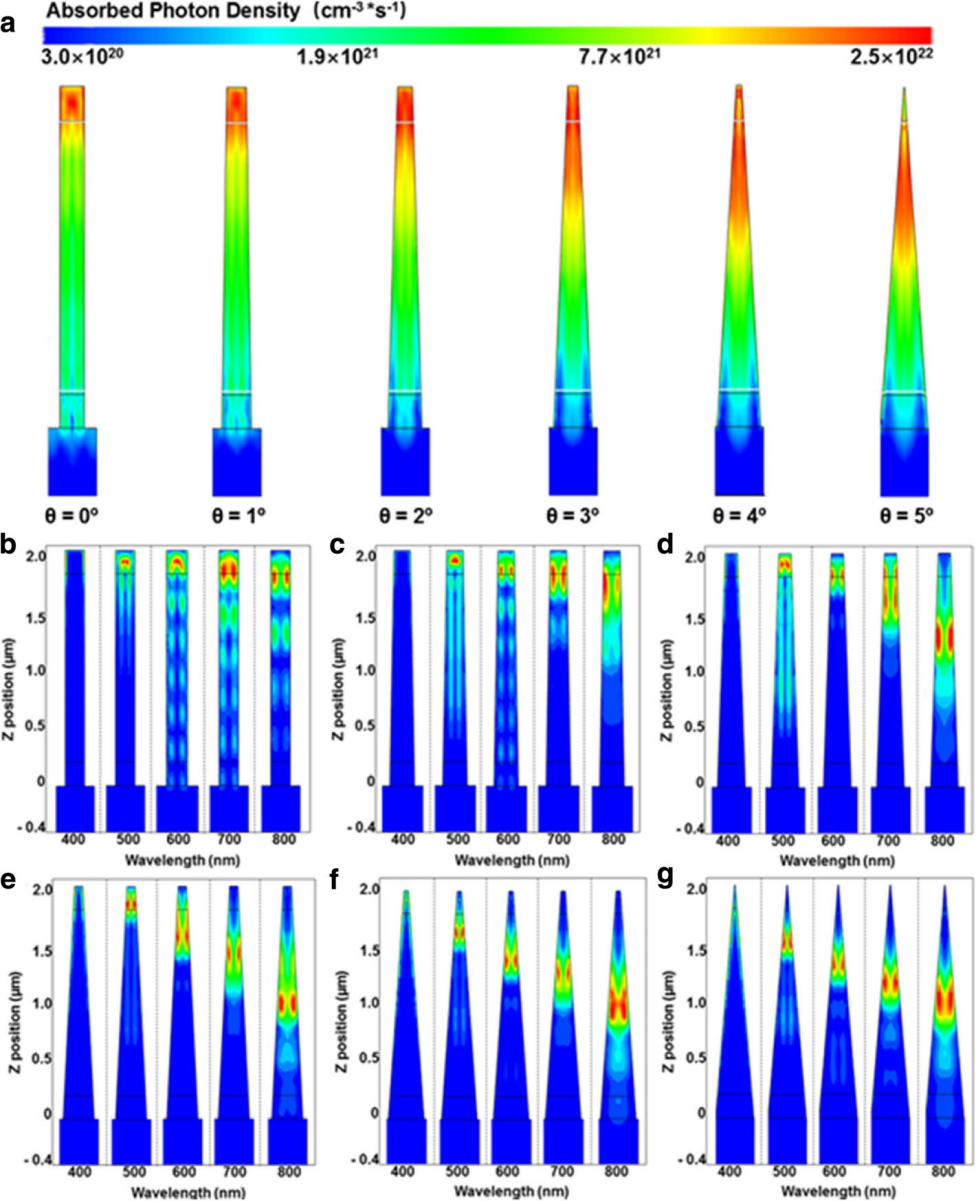
**a** The total optical generation profiles of the axial pin nanocones. **b**–**g** Wavelength-dependent optical generation profiles of nanocone arrays at *θ* = 0~ 5°

The total optical generation profiles of the axial GaAs nanocone arrays under AM 1.5G illumination are shown in Fig. 3a. It can be seen that at *θ* = 0°, most of the absorbed photons concentrate at the top of cylinder NWs. Due to the high doping concentration and lack of built-in electric field for the separation of electron-hole pairs [34–37], the recombination of photocarriers in the top p-region is very high, resulting in large loss of incident light. For nanocone arrays, the photon absorption position shifts downward with the increase of slope angle, leading to an absorption enhancement in the i-region. As has been reported, the light absorption of NWs is dominated by the resonant modes, which are closely related to the NW diameter [37]. Due to the unique geometry of nanocones, few long wavelength modes can be supported in the top region with a small diameter. This is supported by Fig. 3b–g, which present the wavelength-dependent optical generation profiles of nanocones with a slope angle of 0~ 5°. It can be seen that in cylinder NWs, most of the absorption concentrates at the top region for all wavelengths. However, as the slope angle increases, the optical modes, particularly the longer wavelength modes, shift downward to a thicker region. Hence, the increase of slope angle not only leads to an absorption enhancement in the middle i-region but also results in an absorption reduction in the top region. This can explain why the nanocone array with a mediate slope angle of 3° has the high total absorption as shown in Fig. 3e, as the absorption in both the top p-region and middle intrinsic regions is relatively strong at that angle. It is believed that the downward shift of the absorption plays a critical role in the performance improvement of the device as it not only suppresses the absorption loss in the top p-region but also enhances the absorption in the middle i-region.

**Fig. 4:**
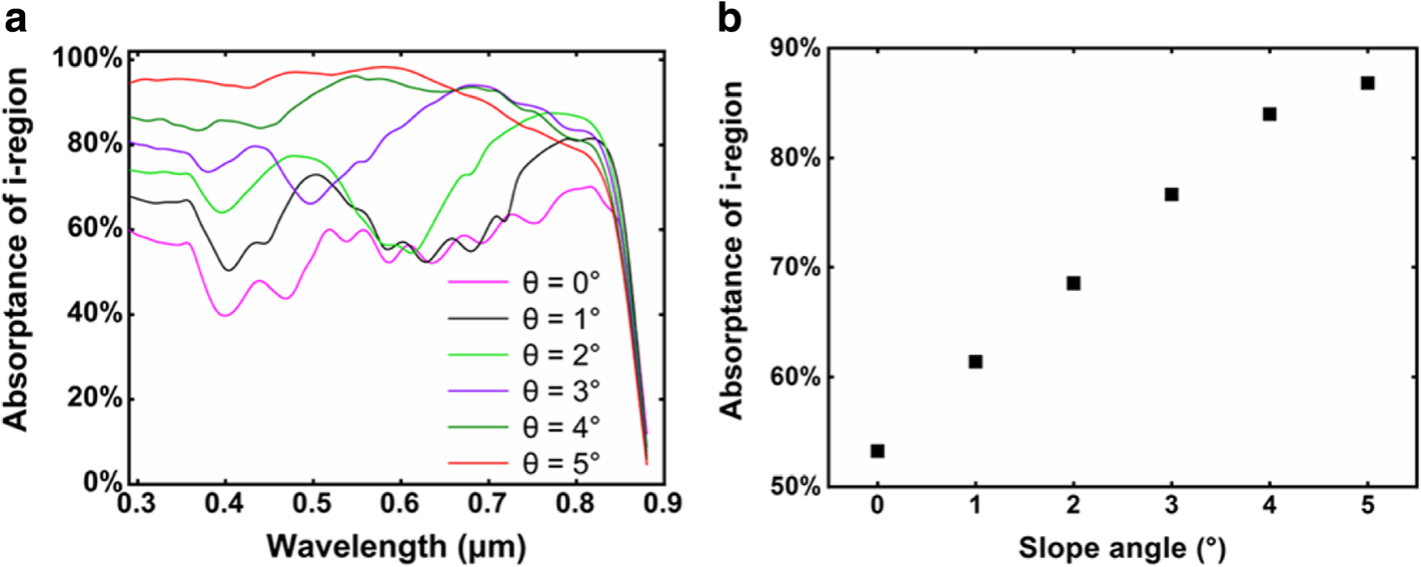
**a** Wavelength-dependent absorption spectra of the i-region. **b** The AM1.5G-weighted integral of the absorption spectra of i-region in **a**

The absorption spectra of the i-region are plotted in Fig. 4a. At short wavelength region, as the diameter of the p-region shrinks with increasing slope angle, both the p-region volume and the light power that can be confined in the nanocone decrease, leading to insufficient absorption in the p-region and high absorptance in the i-region. At long wavelength region, the absorption region extends into the bottom n-region in nanocones at large slop angle, resulting in decreased absorptance in the i-region. Figure 4b shows the integral of the absorption spectra in i-region. The absorptance of each wavelength is weighted by the AM 1.5G spectrum. It can be seen that the absorption in i-region dramatically increases with increasing the slope angle, indicating an enhanced effective absorption which is expected to improve the conversion efficiency.

**Fig. 5:**
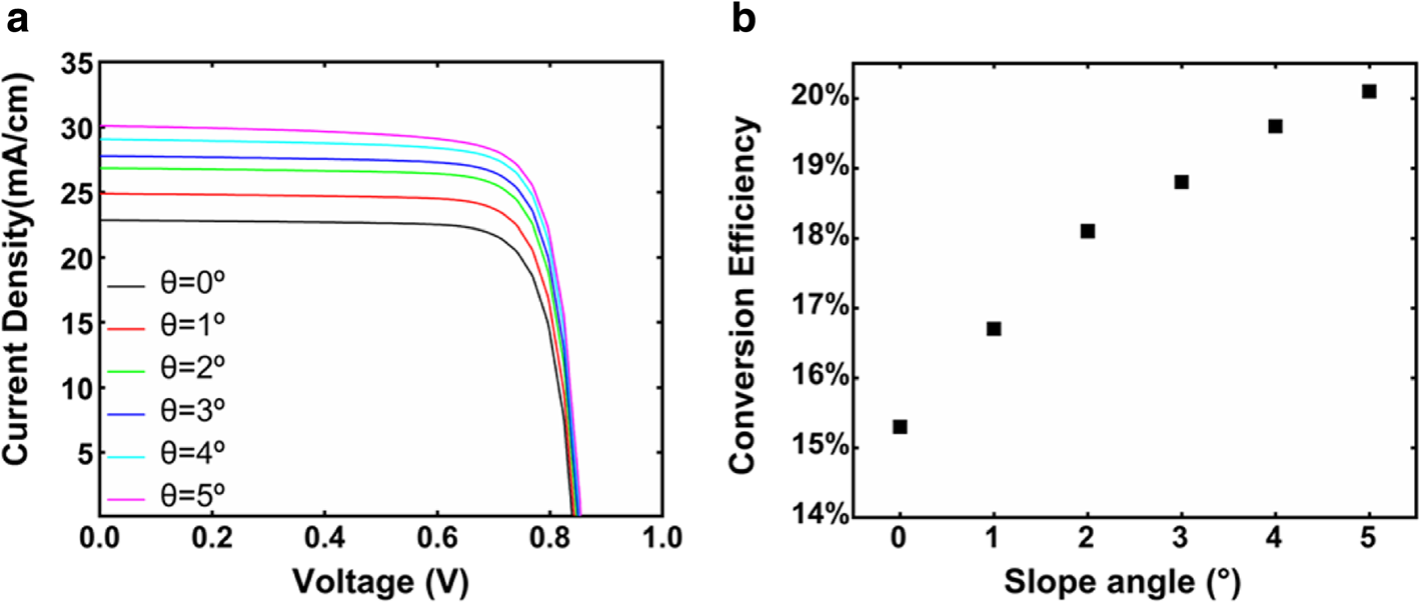
**a** Current-voltage curves of axial p(i)n junction nanocone array solar cells with different slope angles. **b** Photoelectric conversion efficiency of axial p(i)n junction nanocone array solar cells with different slope angles

The photogeneration profiles are then incorporated into the electrical tool [35] to study the photovoltaic performance of the axial pin junction nanocone array solar cell. Figure 5a shows the current-voltage characteristics at different slope angles. Compared with the cylinder NW arrays, much higher short-circuit current density (*J*_sc_) is obtained in nanocone array solar cells. At *θ* = 5°, the device yields a *J*_sc_ of 30.1 mA/cm^2^ (7.3 mA/cm^2^ higher than the cylinder one) and *V*_oc_ of 0.885 V, resulting in a high photoelectric conversion efficiency (*η*) of 20.1% (4.8% higher than the cylinder one). Figure 5b plots the dependence of conversion efficiency on the slope angle. As the slope angle increases from 0 to 5°, the conversion efficiency monotonically increases from 15.3 to 20.1%. As mentioned earlier, the absorption of the whole nanocone arrays saturates at *θ* = 2°, suggesting that the efficiency enhancement at large slope angle is not caused by the absorption enhancement of the whole nanocone arrays. Instead, the trend of the conversion efficiency is highly in accordance with the absorption in i-region shown in Fig. 4b, demonstrating that the conversion efficiency is dominated by the effective optical absorption in i-region.

### Radial Pin Junction GaAs Nanocone Array Solar Cells

**Fig. 6:**
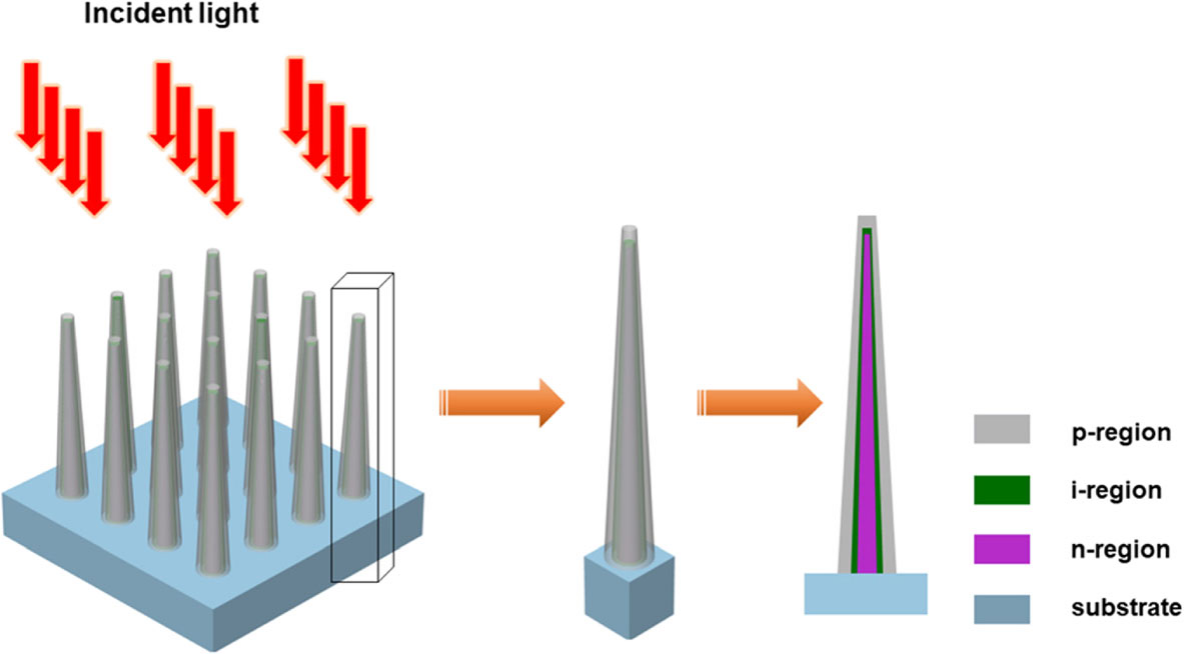
The schematic diagram of the radial pin junction GaAs nanocone arrays

The radial pin junction GaAs nanocone array model is shown in Fig. 6, which consists of periodic radial pin GaAs nanocones with diameter *D* = 360 nm, period *P* = 720 nm, and length *L* = 2 μm. The thickness of the i-region is 10 nm, and the radius of the core equals the shell thickness. The doping concentrations of the n-type core and p-type shell are set to be the same with that of the axial nanocones. The slope angle is changed from 0 to 10° by varying the bottom and top diameter while maintaining the average diameter constant.

**Fig. 7:**
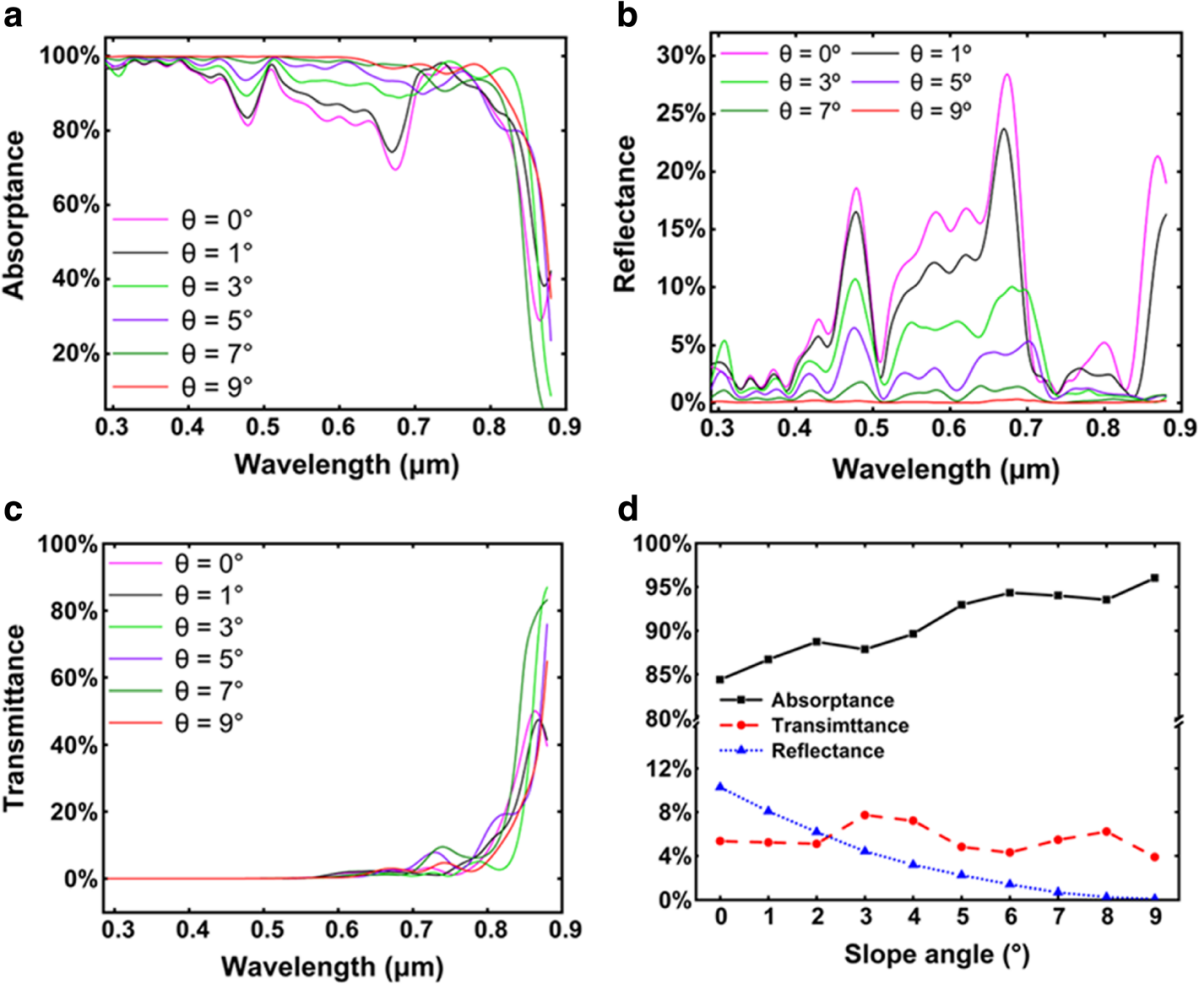
**a** Absorptance, **b** reflectance, and **c** transmittance of the radial pin junction GaAs nanocone array with *D*/*P* = 0.5 and *D* = 0.36 μm. **d** The AM1.5G-weighted integral of the absorptance, reflectance, and transmittance of the radial nanocone array with different slope angles

The wavelength-dependent absorptance, reflectance, and transmittance of radial GaAs nanocone arrays with different slope angles are shown in Fig. 7a–c. Similar to axial structures, radial nanocons show lower reflectance over the whole wavelength range compared to the radial cylinder NWs (*θ* = 0°), and this phenomenon becomes more obvious with the increase of slope angle. In Fig. 7a, it can be seen that in the short wavelength range of 300–700 nm, the absorptance increases with the increase of slope angle due to the suppression of reflectance. While at large slope angles, the nanocone top is too thin to support long wavelength modes, resulting in a decrease of the absorptance. Figure 7d shows the AM 1.5G-weighted integral of the absorptance, reflectance, and transmittance spectra for different slope angles. It can be seen that as the slope angle increases, the absorption exhibits an overall upward trend with slight fluctuations, suggesting excellent absorption properties for nanocone structures.

**Fig. 8:**
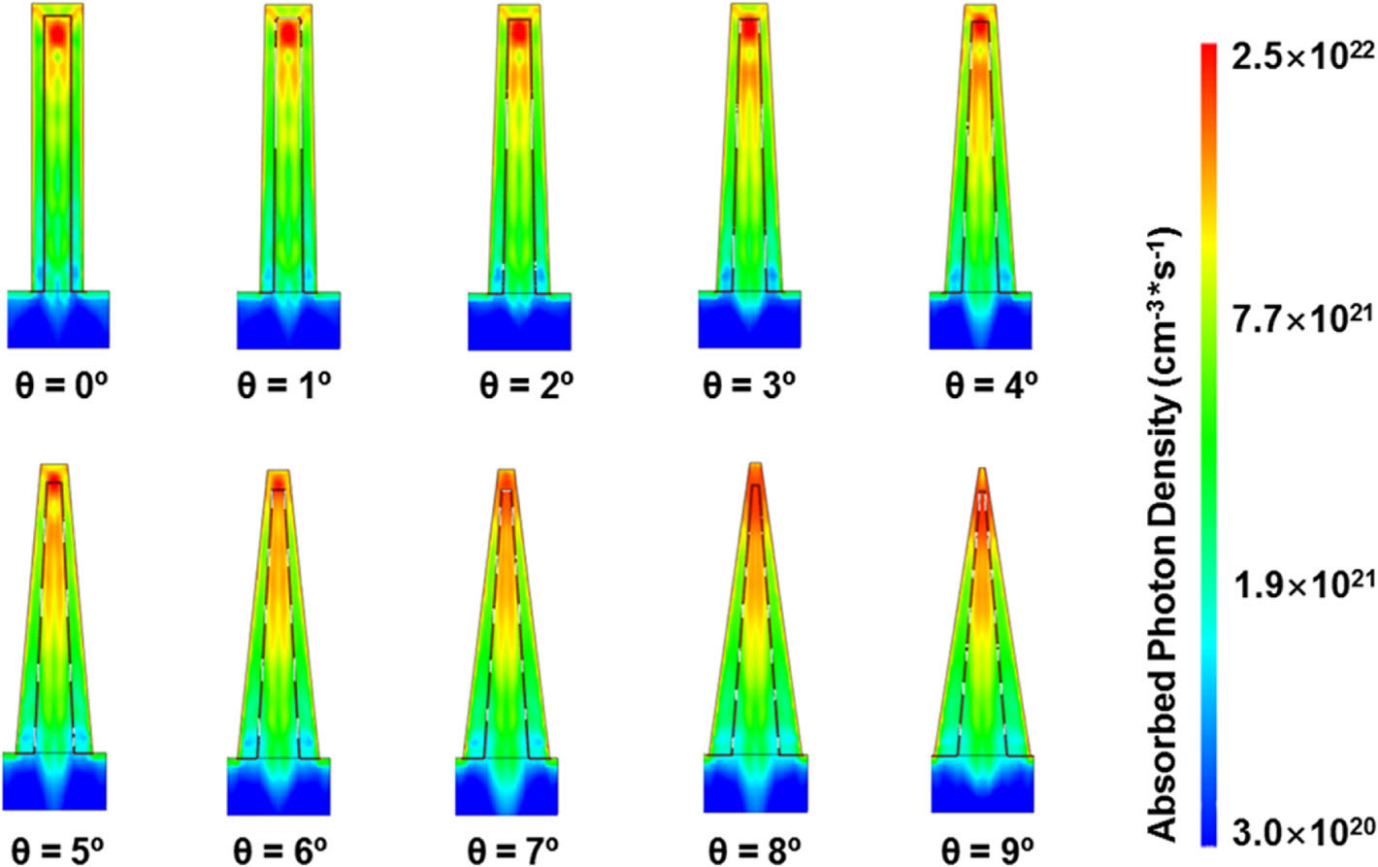
The optical generation profiles of the radial pin nanocone arrays with different slope angles

Figure 8 shows the total optical generation profiles of the radial GaAs nanocone arrays under AM 1.5G illumination. Similar with that in axial arrays, most of the photons concentrate at the top of cylinder NWs. As the slope angle gradually increases, the absorption shifts downward. As the i-region tube in the radial junction penetrates the whole NW, the downward shift of absorption cannot directly leads to an absorption enhancement as that in the axial pin junction. However, together with the downward shift of absorption, the absorption length also extends, resulting in an enhanced overlap between the light absorption and i-region. Hence, the effective absorption is also believed to be enhanced.

**Fig. 9:**
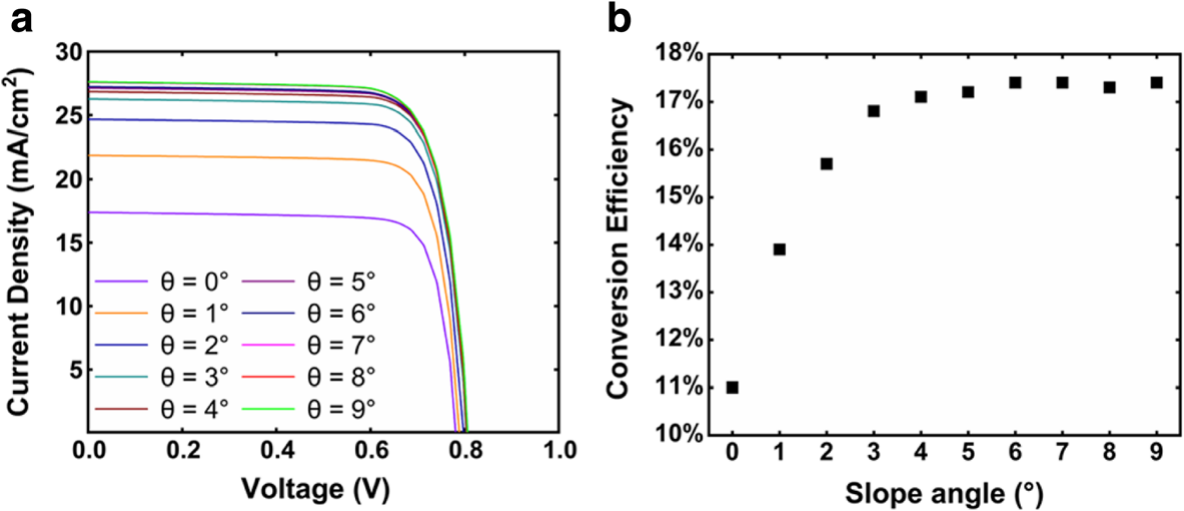
**a** Current-voltage curves of radial pin junction nanocone array solar cells with different slope angles. **b** Dependence of the conversion efficiency on the slope angle

The current-voltage characteristics of the radial nanocone solar cells are shown in Fig. 9a. Compared with the cylinder NW array solar cell, much higher *J*_sc_ is achieved in nanocone array solar cells. At *θ* ≥ 6°, all the *J*_sc_ exceed 25 mA/cm^2^, by contrast, the *J*_sc_ is 17.4 mA/cm^2^ at *θ* = 0°. Figure 9(b) shows the dependence of conversion efficiency on the slope angle. At small slope angles, the efficiency monotonically increases with the slope angle and reaches a maximum value of 17.4% at *θ* = 6°, 6.4% higher than the cylinder counterpart. When the angle further increases, the efficiency saturates and even slightly decreases. This is probably attributed to the competition between the absorption of the top and middle i-regions. At a large slope angle, the nanocone top is too thin to support long wavelength modes. Although the absorption of the middle i-region part increases due to the downward shift of absorption, the absorption in the top i-region part decreases, offsetting the absorption increment in the middle i-region.

## Conclusions

In summary, we have studied the photovoltaic performance of axial and radial pin junction GaAs nanocone array solar cells by a coupled 3D optoelectronic simulation. The results show that the absorption in the nanocone array shifts downward due to the shrinking top diameter, which dramatically suppresses the absorption loss in the high-doped top region and enhances the absorption in the depletion region. The highest conversion efficiencies for axial and radial GaAs nanocone solar cells are 20.1% and 17.4%, obtained at a slope angle of 5° and 6°, respectively, both of which are much higher than their cylinder NW counterparts. The nanocone structures are promising candidates for high-efficiency solar cells.

## Abbreviations

3D: Three-dimensional
*D*/*P*: Diameter/period
EMW: Sentaurus Electromagnetic Wave
FDTD: Finite-difference time-domain
FEM: Finite element method
NW: Nanowire
PML: Perfect match layer
SRH: Shockley-Read-Hall

## References

[1] Luque A, Martí A, Stanley C (2012). Understanding intermediate-band solar cells. Nat Photonics.

[2] Garnett E, Yang P (2010). Light trapping in silicon nanowire solar cells. Nano Lett.

[3] Li H, Qin M, Wang L (2017). Total absorption of light in monolayer transition-metal dichalcogenides by critical coupling. Opt Express.

[4] Li HJ, Ren YZ, Hu J, Qin M, Wang L (2018). Wavelength-selective wide-angle light absorption enhancement in monolayers of transition-metal dichalcogenides. J Lightwave Technol.

[5] Li H, Wang L, Zhai X (2016). Tunable graphene-based mid-infrared plasmonic wide-angle narrowband perfect absorber. Sci Rep.

[6] Guo H, Wen L, Li X, Zhao Z, Wang Y (2011). Analysis of optical absorption in GaAs nanowire arrays. Nanoscale Res Lett.

[7] Wu D, Tang X, Wang K, He Z, Li X (2017). An efficient and effective design of InP nanowires for maximal solar energy harvesting. Nanoscale Res Lett.

[8] Convertino A, Cuscuna M, Rubini S, Martelli F (2012). Optical reflectivity of GaAs nanowire arrays: experiment and model. J Appl Phys.

[9] Wang Y, Zhang Y, Zhang D, He S, Li X (2015). Design high-efficiency III-V nanowire/Si two-junction solar cell. Nanoscale Res Lett.

[10] Holm JV, Jørgensen HI, Krogstrup P, Nygård J, Liu H, Aagesen M (2013). Surface-passivated GaAsP single-nanowire solar cells exceeding 10% efficiency grown on silicon. Nat Commun.

[11] LaPierre RR (2011). Theoretical conversion efficiency of a two-junction III-V nanowire on Si solar cell. J Appl Phys.

[12] Borgström MT, Magnusson MH, Dimroth F, Siefer G, Höhn O, Riel H, Schmid H, Wirths S, Björk M, Åberg I, Peijnenburg W, Vijver M, Tchernycheva M, Piazza V, Samuelson L (2018) Towards nanowire tandem junction solar cells on silicon. IEEE J Photovoltaics 8(3):733–40.

[13] Heurlin M, Wickert P, Fält S, Borgström MT, Deppert K, Samuelson L, Magnusson MH (2011). Axial InP nanowire tandem junction grown on a silicon substrate. Nano Lett.

[14] Åberg I, Vescovi G, Asoli D, Naseem U, Gilboy JP, Sundvall C, Dahlgren A, Svensson KE, Anttu N, Björk MT, Samuelson L (2015). A GaAs nanowire array solar cell with 15.3% efficiency at 1 sun. IEEE J Photovoltaics.

[15] Mariani G, Zhou Z, Scofield A, Huffaker DL (2013). Direct-bandgap epitaxial core-multishell nanopillar photovoltaics featuring subwavelength optical concentrators. Nano Lett.

[16] Kupec J, Stoop RL, Witzigmann B (2010). Light absorption and emission in nanowire array solar cells. Opt Express.

[17] Jäger ST, Strehle S (2014). Design parameters for enhanced photon absorption in vertically aligned silicon nanowire arrays. Nanoscale Res Lett.

[18] Gu Z, Prete P, Lovergine N, Nabet B (2011). On optical properties of GaAs and GaAs/AlGaAs core-shell periodic nanowire arrays. J Appl Phys.

[19] Anttu N, Abrand A, Asoli D, Heurlin M, Åberg I, Samuelson L, Borgström M (2014). Absorption of light in InP nanowire arrays. Nano Res.

[20] Wen L, Zhao Z, Li X, Shen Y, Guo H, Wang Y (2011). Theoretical analysis and modeling of light trapping in high efficicency GaAs nanowire array solar cells. Appl Phys Lett.

[21] Wu Y, Yan X, Zhang Z, Ren X (2015). Enhanced photovoltaic performance of an inclined nanowire array solar cell. Opt Express.

[22] Joyce HJ, Gao Q, Tan HH, Jagadish C, Kim X, Zhang X, Guo Y (2007). Twin-free uniform epitaxial GaAs nanowires grown by a two-temperature process. Nano Lett.

[23] Soci C, Bao XY, Aplin DPR, Wang D (2008). A systematic study on the growth of GaAs nanowires by metal−organic chemical vapor deposition. Nano Lett.

[24] Chuang LC, Moewe M, Ng KW, Tran TTD, Crankshaw S, Chen R, Ko WS, Chang-Hasnaina C (2011). GaAs nanoneedles grown on sapphire. Appl Phys Lett.

[25] Chuang LC, Sedgwick FG, Chen R, Ko WS, Moewe M, Ng KW, Tran TTD, Chang-Hasnaina C (2010). GaAs-based nanoneedle light emitting diode and avalanche photodiode monolithically integrated on a silicon substrate. Nano Lett.

[26] Wang B, Stevens E, Leu PW (2014). Strong broadband absorption in GaAs nanocone and nanowire arrays for solar cells. Opt Express.

[27] Fountaine KT, Kendall CG, Atwater HA (2014). Near-unity broadband absorption designs for semiconducting nanowire arrays via localized radial mode excitation. Opt Express.

[28] Xie WQ, Liu WF, Oh JI, Shen WZ (2011). Optical absorption in c-Si/a-Si: H core/shell nanowire arrays for photovoltaic applications. Appl Phys Lett.

[29] Zanuccoli M, Semenihin I, Michallon J, Sangiorgi E, Fiegna C (2013). Advanced electro-optical simulation of nanowire-based solar cells. J Comput Electron.

[30] Huang N, Lin C, Povinelli ML (2012). Limiting efficiencies of tandem solar cells consisting of III-V nanowire arrays on silicon. J Appl Phys.

[31] Wen L, Li X, Zhao Z, Bu S, Zeng X, Huang JH, Wang Y (2012). Theoretical consideration of III-V nanowire/Si triple-junction solar cells. Nanotechnology.

[32] Levinshtein M, Rumyantsev S, Shur M (1996). Handbook Series on Semiconductor Parameters.

[33] Li Y, Yan X, Wu Y, Zhang X, Ren X (2015). Plasmon-enhanced light absorption in GaAs nanowire array solar cells. Nanoscale Res Lett.

[34] Roulston DJ, Arora ND, Chamberlain SG (1982). Modeling and measurement of minority-carrier lifetime versus doping in diffused layers of n+-p silicon diodes. IEEE T Electron Dev.

[35] Fossum JG, Lee DS (1982). A physical model for the dependence of carrier lifetime on doping density in nondegenerate silicon. Solid State Electron.

[36] Fossum JG, Mertens RP, Lee DS, Nijs JF (1983). Carrier recombination and lifetime in highly doped silicon. Solid State Electron.

[37] Nicklas A, Xu HQ (2010). Coupling of light into nanowire arrays and subsequent absorption. J Nanosci Nanotechnol.

